# Protecting the public interest when regulating health professionals providing virtual care: a scoping review protocol

**DOI:** 10.1186/s13643-023-02198-1

**Published:** 2023-03-06

**Authors:** Kathleen Leslie, Sophia Myles, Tracey L. Adams, Catharine Schiller, Jacob Shelley, Sioban Nelson

**Affiliations:** 1 Faculty of Health Disciplines, Athabasca University, 1 University Drive, Athabasca, AB T9S 3A3 Canada; 2 Department of Sociology, Western University, London, ON Canada; 3 School of Nursing, University of Northern British Columbia, Prince George, BC Canada; 4 Faculty of Law and School of Health Studies, Western University, London, ON Canada; 5 Faculty of Nursing, University of Toronto, Toronto, ON Canada

**Keywords:** Patient safety, Professional regulation, Public interest, Telehealth, Virtual care, Licensure, Telemedicine

## Abstract

**Background:**

Virtual care is transforming the nature of healthcare, particularly with the accelerated shift to telehealth and virtual care during the COVID-19 pandemic. Health profession regulators face intense pressures to safely facilitate this type of healthcare while upholding their legislative mandate to protect the public. Challenges for health profession regulators have included providing practice guidance for virtual care, changing entry-to-practice requirements to include digital competencies, facilitating interjurisdictional virtual care through licensure and liability insurance requirements, and adapting disciplinary procedures. This scoping review will examine the literature on how the public interest is protected when regulating health professionals providing virtual care.

**Methods:**

This review will follow the Joanna Briggs Institute (JBI) scoping review methodology. Academic and grey literature will be retrieved from health sciences, social sciences, and legal databases using a comprehensive search strategy underpinned by Population-Concept-Context (PCC) inclusion criteria. Articles published in English since January 2015 will be considered for inclusion. Two reviewers will independently screen titles and abstracts and full-text sources against specific inclusion and exclusion criteria. Discrepancies will be resolved through discussion or by a third reviewer. One research team member will extract relevant data from the selected documents and a second will validate the extractions.

**Discussion:**

Results will be presented in a descriptive synthesis that highlights implications for regulatory policy and professional practice, as well as study limitations and knowledge gaps that warrant further research. Given the rapid expansion of virtual care provision by regulated health professionals in response to the COVID-19 pandemic, mapping the literature on how the public interest is protected in this rapidly evolving digital health sector may help inform future regulatory reform and innovation.

**Systematic review registration:**

This protocol is registered with the Open Science Framework (10.17605/OSF.IO/BD2ZX).

**Supplementary Information:**

The online version contains supplementary material available at 10.1186/s13643-023-02198-1.

## Background

Virtual care is increasingly transforming the nature of healthcare, particularly with the rapid shift to telehealth and virtual care during the COVID-19 pandemic. Health profession regulators face intense pressure to facilitate virtual care while upholding their legislative mandate to protect the public. However, there are many legal and ethical complexities associated with regulating registrants who engage in virtual care. Regulatory activities in the public interest have changed to encompass the following: ensuring that standards provide necessary guidance for virtual care on topics such as consent, documentation, records management, and privacy [1, 2]; changing entry-to-practice requirements to include digital competencies; facilitating interjurisdictional virtual care through reciprocal licensure, emergency licensure limited to virtual service provision, and liability insurance requirements [3, 4]; and adapting continuing competence requirements and disciplinary procedures to reflect modern digital environments [5]. The need to reform regulatory practices to meet these challenges to ensure access to safe and high-quality services has become more urgent given the impact of the COVID-19 pandemic on health care and health professionals.

The mandate of health profession regulators is to protect patients and uphold the public interest. Regulators typically aim to meet this public protection mandate by setting entry-to-practice standards; maintaining a register of those who are licensed to practice; and monitoring and enforcing conduct, competency, and capacity in practice [6]. The goal is to protect the public against incompetence, negligence, and dishonesty by ensuring only those fit to practice safely are registered [7]. However, there has been debate and controversy about which activities should be included under this public interest mandate, and practices have varied over time [8, 9].

Virtual care is not new, and over the last decade, there has been increased reliance on virtual care globally [4]. However, the uptake of virtual care was accelerated by the COVID-19 pandemic. A study in Ontario, Canada, found that virtual care use rose across the population, increasing from 1.6% of total ambulatory visits in the second quarter of 2019 to 70.6% in the second quarter of 2020 [10]. This data is consistent with that coming out of the USA, where one study found that the uptake of virtual care increased from 4% before the pandemic to 35% during the pandemic [10]. This rapid scaling of virtual care is a major factor that distinguishes the regulatory response to the COVID-19 pandemic from previous public health crises or emergencies. As a result, health profession regulators face unprecedented pressure to facilitate this transition safely and effectively.

Critical to regulating virtual care is the issue of cross-jurisdictional practice. In Canada, where there is much variability in the approach to health profession regulation across provincial and territorial borders, this has been a significant barrier to equitable access to care during the COVID-19 pandemic. In the USA, where regulation is similarly under state jurisdiction, reforms have facilitated cross-jurisdictional practice during the pandemic, including modifying many in-state licensure requirements for telehealth [3] and relying on licensure compacts to enable telehealth [11]. In Canada, despite some regulatory reform around virtual care, variations in licensure requirements and scopes of practice, as well as difficulties ascertaining to which regulator professionals are accountable, have continued to complicate virtual cross-jurisdictional care [12–14].

Many reforms related to regulating virtual care were fast-tracked, but calls have already been made for regulatory frameworks to be subject to careful post-implementation reviews—a means to ensure the public is protected [15]. Others have argued that relaxing regulatory barriers during the pandemic (for example, expanding nursing scope of practice to address crisis surge capacity needs) demonstrates that these requirements were unnecessary for public protection in the first place [16] and have called for these changes to be retained even after the crisis subsides [17, 18]. Whether these regulatory reforms contribute to public protection in the short, medium, and long term requires an understanding of the literature regarding regulating virtual care in the public interest.

The literature around regulating virtual care is diverse and spans health, social sciences, and law. A preliminary search of PROSPERO, MEDLINE, the Cochrane Database of Systematic Reviews, and JBI Evidence Synthesis was conducted and found no existing scoping reviews or systematic reviews on the topic. A scoping review will generate a broad overview of this topic to identify and summarize the range of evidence available.

## Review question and objective

Our research question is: *How is the public interest protected when regulating health professionals engaged in virtual care?* The objective of this review is to examine the literature on how the public interest is protected when regulating health professionals engaged in virtual care, to discuss policy and practice implications related to health professional regulation of virtual care, and to make recommendations for future research.

## Methods

### Study design

This scoping review will follow JBI methodology for scoping reviews [19], including the most recent guidance [20], and will use the reporting guidelines provided in the Preferred Reporting Items for Systematic Reviews and Meta-analyses extension for Scoping Reviews (PRISMA-ScR) checklist [21]. This protocol is registered with the Open Science Framework (10.17605/OSF.IO/BD2ZX). This protocol has been reported with the reporting guidelines in the PRISMA extension for protocols (PRISMA-P) [22] as they apply to scoping reviews (see PRISMA-P checklist in Additional file 1).

### Eligibility criteria

We have used the Population-Concept-Context (PCC) approach recommended by the JBI scoping review methodology to define our eligibility criteria.

#### Population

This review will consider studies that involve any regulated health professionals. In this protocol, regulated health professionals are those healthcare providers governed by regulatory bodies with the legal mandate to protect the public, such as nursing, medicine, pharmacy, and psychology.

#### Concept

This review will consider studies that discuss protecting the public interest when regulating health professionals engaged in virtual care. Protecting the public may also be described as ensuring patient or client safety, though the public interest is often conceptualized more broadly in the Canadian context. Virtual care includes any form of digitally enabled healthcare practice such as telehealth or other virtual care delivery with no in-person interaction.

#### Context

This review will consider studies that discuss protecting the public interest when regulating health professionals providing virtual care in any country or setting. Functions of regulatory bodies that contribute to public protection include setting entry-to-practice and registration requirements, monitoring conduct and competency in practice, and sanctioning negligence or misconduct by individual professionals. We will exclude articles focused on other types of government or industry regulation such as pharmaceuticals, medical devices, or provider reimbursement.

### Types of sources

We will consider any type of study design for inclusion in this review. English-language grey literature in the public domain that specifically discusses regulating health professionals providing virtual care (such as legal briefs, government reports, documents from regulatory consortiums, and policy papers) will also be considered for inclusion. We will exclude practice guidance or standards from individual health profession regulators (given the volume of documents this would represent); we will also exclude editorials, letters to the editor, textbook chapters, and conference abstracts.

### Search strategy

The literature around professional regulation of virtual care is diverse and it is important to capture various disciplinary perspectives through a comprehensive search strategy. As such, we will search academic databases that cover *health science literature* (MEDLINE [Ovid], and Embase [Ovid]), *social science literature* (Sociological Abstracts [Proquest], Social Work Abstracts [EBSCO]), and *legal literature* (HeinOnline, CanLII, and WestlawNext Canada) as well as the interdisciplinary database Scopus and search engine Google Scholar. In addition, we will conduct an iterative grey literature search including grey literature databases (OpenGrey, Nexis Uni, ProQuest Dissertations, and Theses Global), search engines (first 200 Google results), and relevant websites (e.g., COVID-19 Law Lab, OECD Regulatory Policy Division, GovInfo, and Government of Canada) based on keywords used in the academic database searches and in consultation with the research team.

We conducted an initial limited search of the Scopus database to identify a selection of relevant articles on the topic. The research team reviewed the titles and abstracts and index terms used to describe the articles to develop a list of keywords. The search strategy will then be adapted across the various databases in consultation with a research librarian. The search will cover papers published in English from 1 January 2015. This date range will allow us to feasibly capture the most recent evidence given the rapidly evolving nature of telehealth and virtual care, particularly during the COVID-19 pandemic. A full search strategy for MEDLINE (Ovid) is provided (see Additional file 2).

### Study selection

Following the search, all identified records will be collated and uploaded into Covidence (https://www.covidence.org) and duplicates removed. Following a pilot test, titles and abstracts will be screened by two independent reviewers against the inclusion and exclusion criteria for the review. Two independent reviewers will assess the full text of selected citations. Reasons for exclusion of full-text papers that do not meet the inclusion criteria will be recorded and reported in the scoping review final report. Any disagreements that arise between the reviewers will be resolved through discussion or with a third reviewer.

### Data extraction

Data will be extracted from sources included in the scoping review using a modification of the JBI data extraction tool developed for this scoping review by the research team. The data extracted will include specific details about the population (e.g., the specific regulated health profession), the concept of regulating health professionals working in virtual care, and the context (e.g., jurisdiction and specific regulatory activities), as well as key findings relevant to the review objectives and question and reviewer comments. Draft extraction tools for both academic and grey literature are provided (see Additional file 3). The data extraction form will be piloted for 10 sources of academic literature and 10 sources of grey literature. The data extraction form will be continuously revised in an iterative process throughout the data charting as we become more familiar with the literature. Extracted data will be managed through the Covidence Extraction 2.0 tool. One reviewer will extract data from each source and a second reviewer will verify the extraction. Any disagreements that arise between the reviewers will be resolved through discussion or with a third reviewer. Authors of papers will be contacted to request missing or additional data, where required.

### Data presentation and analysis of results

The results will be presented in figures, tables, and text as appropriate based on the extracted data. The search results and the study selection process will be reported in the final scoping review and presented in a PRISMA flow diagram. We will include a descriptive analysis of the characteristics of the included studies, including types of papers and study design, geographic focus or context, and practitioner groups, disciplines, or specialties. Due to the nature of this topic, we anticipate many included sources will be critical commentaries, legal analyses, or qualitative studies, and anticipate grey literature sources will feature prominently as well. As such, the main results will be presented in a narrative description by summarizing and synthesizing the extracted data according to key findings. However, this synthesis methodology may be refined based on the types of studies included and the data extracted. Based on our review objectives, we will highlight implications for regulatory policy and professional practice, as well as study limitations and knowledge gaps that warrant further research.

## Discussion

Given the rapid expansion of virtual care provision by regulated health professionals in response to the COVID-19 pandemic, mapping the literature on how the public interest is protected in this rapidly evolving digital health sector may help inform future regulatory reform and innovation. New and disruptive technologies in virtual care will likely have a continued relevance for practitioners and regulators will need to grapple with these emerging practice issues to ensure guidance and standards are fit for purpose. Recently, policymakers and researchers have pushed for regulatory reform to be based on current evidence [23, 24]. Thus, the findings of this review may be of interest to policymakers, regulators, and researchers, particularly as jurisdictions begin to focus on post-pandemic recovery and new ways of ensuring equitable access to health care, including via virtual means. We will publish the findings of the review open-access and disseminate key results to stakeholders via webinars and presentations to increase the uptake of insights from this review.

This scoping review is limited to English-language sources published since 2015, so we may miss earlier studies or those in other languages that could inform our review. However, given language limitations of the research team, the rapidly evolving nature of this topic, and results of our initial searches, we deemed these limitations necessary to feasibly complete this review. Because this is a scoping review, we will not be conducting a formal evaluation of quality or bias of the included articles, representing another limitation. Nonetheless, to our knowledge, this is the first scoping review about protecting the public interest when regulating health professionals providing virtual care. This scoping review will inform future research that will give more depth and breadth to these findings.

## Supplementary Information

**Additional file 1.** PRISMA-P 2015 Checklist.

**Additional file 2.** Search strategy.

**Additional file 3.** Data extraction templates.

## Data Availability

Not applicable.

## Abbreviations

CINAHL: Cumulative Index to Nursing and Allied Health Literature
JBI: Joanna Briggs Institute
PCC: Population-Concept-Context
PRISMA-ScR: Preferred Reporting Items for Systematic Reviews and Meta-Analyses extension for scoping reviews
PRISMA-P: Preferred Reporting Items for Systematic Reviews and Meta-Analyses extension for protocols
