# Supplementary material for: Protecting the public interest when regulating health professionals providing virtual care: a scoping review protocol
Source: Syst Rev. 2023 Mar 6;12:31. doi: 10.1186/s13643-023-02198-1 (PMC9986861; doi:10.1186/s13643-023-02198-1)
Supplement: Supplementary file 2 — Additional file 2. Search strategy. [file 13643_2023_2198_MOESM2_ESM.docx]

# Additional file 2: Search strategy

Search strategy for MEDLINE via Ovid conducted on 29 May 2021.

| **Search** | **Query** | **Records Retrieved** |
| --- | --- | --- |
| 1 | exp telemedicine/ or internet-based intervention/ or ((videoconferencing/ or electronic mail/ or exp cell phone/ or exp internet/ or telephone/ or mobile applications/) and (exp patient care management/ or exp patient care/)) | 49381 |
| 2 | (telehealth or telecaring or telecare or telemedicine or telenurs* or telerehab* or teleconsult* or mhealth or ehealth or mobile health or telepathol* or teleradiol* or telemonitor* or tele monitor* or telediagnos* or telepsych* or erehab* or telesurger* or teletherap* or "m health" or "e health" or teleconferenc* or telecounsel*).ti,ab,kf | 43700 |
| 3 | ((tele or remote or digital or virtual or cyber or online or web or internet or video* or mobile or smartphone or text or texting or telephon* or phone or computer or distant or distance or offsite or app or apps) adj2 (rehab* or consult* or care or caring or healthcare or medical or medicine or nurs* or diagnos* or pathol* or psych* or radiol* or surger* or therap* or specialist* or counsel* or appointment* or visit*)).ti,ab,kf | 49552 |
| 4 | or/1-3 | 110669 |
| 5 | government regulation/ or professional autonomy/ or "facility regulation and control"/ or social control, formal/ or exp jurisprudence/ or exp credentialing/ or exp "legislation as topic"/ | 430853 |
| 6 | exp health services administration/lj or exp occupational groups/lj or (1 and lj.fs) | 76364 |
| 7 | (legislat* or regulations or regulator* or jurispruden* or statutor*).ti | 123779 |
| 8 | (exp patient safety/ or ((patient* or client* or public) adj3 (safety or protect* or interest or interests)).ti,ab,kf.) and (legislat* or regulations or regulator* or jurispruden* or statutor*).ab,kf | 5682 |
| 9 | (profession* adj2 (registration or licens* or licenc* or regulat* or accreditat* or credential* or liab* or negligen* or misconduct or malpractice or college* or board)).ti,ab,kf | 4388 |
| 10 | or/5-9 | 580266 |
| 11 | 4 and 10 | 4909 |
| 12 | limit 11 to yr="2015 -Current" | 1792 |
| 13 | limit 12 to english language | 1705 |
