# Supplementary material for: Protecting the public interest when regulating health professionals providing virtual care: a scoping review protocol
Source: Syst Rev. 2023 Mar 6;12:31. doi: 10.1186/s13643-023-02198-1 (PMC9986861; doi:10.1186/s13643-023-02198-1)
Supplement: Supplementary file 3 — Additional file 3. Data extraction templates. [file 13643_2023_2198_MOESM3_ESM.docx]

# Additional file 3: Data extraction templates

Academic literature extraction template

| Authors (year): |
| --- |
| Research purpose/question(s): |
| Type of research: quantitative, qualitative, mixed methods, review (type), case report, expert report, commentary, observational |
| Population: describe the particular population focus (e.g., the regulated profession(s)) |
| Context: |
| - Jurisdiction |
| - Professional regulatory body and/or specific regulatory activities |
| - Legislative/regulatory framework |
| Concept: |
| - How does this study describe the concept of protecting the public interest when regulating professionals working in virtual practice? (Describe) |
| - What terms or definitions are used to describe concepts such as public interest and virtual practice? (Provide definition or language used by author(s)) |
| - What regulatory activities are discussed? (Describe) |
| Gaps in knowledge/recommendations for further research |
| Reviewer comments: |

Grey literature extraction template

| Authors (year) |
| --- |
| Type of paper: opinion, health policy document, professional guidelines, legal brief, government backgrounder, other (describe) |
| Aim/purpose of the paper: |
| Population: describe the particular population focus (e.g., the regulated profession(s)) |
| Context: |
| - Jurisdiction |
| - Professional regulatory body and/or specific regulatory activities |
| - Legislative/regulatory framework |
| Concept: |
| - How does this document describe the concept of protecting the public interest when regulating professionals working in virtual practice? (Describe) |
| - What terms or definitions are used to describe concepts such as public interest and virtual practice? (Provide definition or language used by author(s)) |
| - What regulatory activities are discussed? (Describe) |
| Recommendations/discussion points: |
| Reviewer comments: |
